# Infective Endocarditis Secondary to Bacteroides Thetaiotaomicron in a Patient With Uterine Smooth Muscle Tumor of Uncertain Malignant Potential: A Case Report and Review of the Literature

**DOI:** 10.7759/cureus.23403

**Published:** 2022-03-22

**Authors:** Rafail Beshai, Ramneet Wadehra

**Affiliations:** 1 Internal Medicine, Jefferson Health - New Jersey, Stratford, USA; 2 Cardiology, Virtua, Camden, USA

**Keywords:** smooth muscle malignancy, aortic endocarditis, endocarditis, thetaiotaomicron, bacteroides

## Abstract

*Bacteroides* species are significant clinical pathogens with an associated mortality of more than 19% and are found in most anaerobic infections. Our report documents for the first time a case of infective endocarditis (IE) secondary to *Bacteroides thetaiotaomicron* (BT). We discuss the case of a 65-year-old female with a medical history of smooth muscle tumor of uncertain malignant potential (STUMP) who presented to the ED with lower quadrant pain. In the hospital, she was found to be in septic shock. A transthoracic echocardiogram showed large vegetation on the aortic valve with severe aortic regurgitation and a blood culture growing BT. We urge physicians to be alert to the fact that Gram-negative anaerobes like BT can cause IE.

## Introduction

*Bacteroides* are Gram-negative obligate anaerobes. They make up approximately 25% of the normal microbiota in the human colon and uterus [1]. Disruption of the mucosal surface by inflammation, cancer, trauma, or surgery can lead to the spread of bacteria into the bloodstream or surrounding tissues, resulting in clinically significant infection [1]. The combination of *Bacteroides thetaiotaomicron *(BT)* *bacteremia, smooth muscle tumor of uncertain malignant potential (STUMP), and infective endocarditis (IE) is very rare. Clinicians should be aware of this unique concurrence for proper diagnosis and management.

## Case presentation

A 65-year-old female with a medical history of metastatic uterine estrogen receptor (ER)/progesterone receptor (PR)-STUMP presented to the ED complaining of lower quadrant pain associated with nausea and vomiting for the past four months. Associated symptoms included chills, fatigue, and weight loss. The patient recently had a biopsy taken in the outpatient setting, which showed cellular proliferation of spindled smooth muscle cells with bland nuclei. Mitotic figures were hard to find. There were focal hemosiderin deposits associated with lymphoplasmacytic infiltrate. The spindle cells were negative for HMB45, ER, PR, and melan-A.

The patient had a temperature of 39.5 °C and a heart rate of 110/minute. Her peripheral blood pressure was 80/40 mmHg. Laboratory exam revealed a total leukocyte count of 22,000/μL. The patient had a flat affect but was answering all questions appropriately. Auscultation revealed a new early diastolic decrescendo murmur heard best at the third intercostal space on the left. Abdominal palpation revealed mild tenderness over her lower abdomen. Norepinephrine bitartrate (Levophed) was administered intravenously to maintain a mean arterial pressure of more than 65 mmHg, and she was admitted to the ICU. Broad-spectrum empiric antibiotics including vancomycin and piperacillin/tazobactam were administered while in the ED.

**Figure 1:**
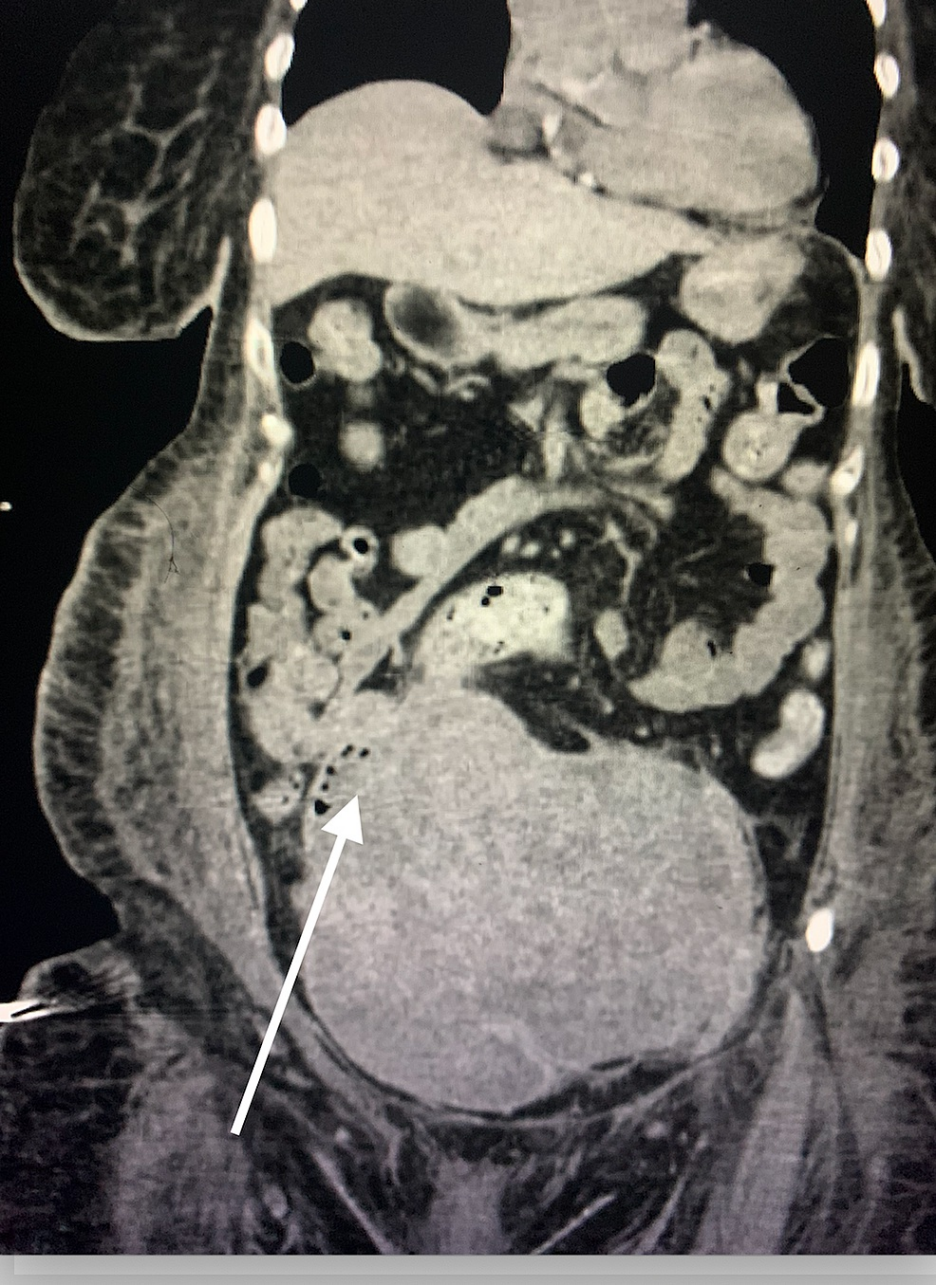
CT scan of abdomen and pelvis showed a large uterine mass with necrotic appearance (white arrow) CT: computed tomography

Upon admission, the ECG showed sinus tachycardia. High-sensitivity troponins were not detected on three serial blood samples. A chest CT scan showed multiple lung nodules concerning for metastases with a small left pleural effusion. A CT angiogram was negative for pulmonary embolism. An abdominal and pelvic CT scan showed a large uterine mass with a necrotic appearance (Figure 1). Two sets of blood cultures were ordered and both grew a Gram-negative bacterium, which was later identified as BT. Given the patient's new murmur, a transthoracic echocardiogram was ordered, which showed large vegetation on her aortic valve (Video 1). It also showed severe aortic regurgitation (Video 2).

**Video 1:** Transthoracic echocardiogram showing large vegetation on the aortic valve (white arrow)

**Video 2:** Transthoracic echocardiogram showing severe aortic regurgitation

The source of the Gram-negative bacterium was believed to be the necrotic uterine mass. The tumor was too enlarged to benefit from chemotherapy. In addition, surgery was not an option as the patient was too unstable for surgery given the large size of the tumor and its intimate involvement with major abdominopelvic vasculature. The patient’s symptoms improved during her extended stay at the hospital. Her blood pressure stabilized without the administration of a vasopressor. Given the diagnosis of end-stage metastatic cancer and since we were unable to control the source of her sepsis, the patient’s prognosis was poor, with less than six months to live. Consequently, the patient was discharged to a subacute rehabilitation facility with hospice care on long-term intravenous piperacillin-tazobactam.

## Discussion

*Bacteroides* maintain a complex and generally beneficial relationship with the host when confined to physiological sites; however, when they escape to sterile locations, they can cause significant pathology, including bacteremia and abscess formation in multiple body sites. BT is one of the most common components of the human gut flora [2]. As with other *Bacteroides *species, some studies have shown that BT is an opportunistic pathogen that can infect tissues under the right conditions, especially in the setting of bacteremia [3].

The incidence of anaerobic bacteremia has been rising since the early 1990s [4]. The mortality rate for bacteremia secondary to *Bacteroides* has been reported to be 16% if active therapy was instituted and 45% if inappropriate therapy was given [5]. About 30% of *Bacteroides* bacteremia is due to underlying malignancy [5]. STUMP is a very rare mesenchymal uterine tumor lying between benign leiomyomas and leiomyosarcomas [6]. Due to its rarity, there is very limited literature on STUMP; there is scant data on its incidence rate and no consensus regarding its histopathological description [7]. BT resides on the endometrium of the human non-pregnant uterus [3]. Consequently, we hypothesize that BT found in our patient’s blood cultures emerged from the tumor.

IE is a serious infectious disease that carries a high risk of morbidity and mortality [8]. The involvement of anaerobic bacteria in endocarditis is very rare due to the high redox potential of the blood [9]. However, serious consequences (including valvular destruction, dysrhythmias, and cardiogenic shock) can happen, with an associated mortality rate of 21-43% [10]. The most common Gram-negative anaerobic bacteria that can cause endocarditis is *Bacteroides fragilis* [9]. Based on our extensive literature review on PubMed and Google Scholar, we believe that this is the first report to describe a case of IE caused by BT.

## Conclusions

This case report highlights the importance of recognizing Gram-negative anaerobes like BT as a cause of IE. It also serves as an alert to clinicians regarding timely detection, diagnosis, and initiation of treatment in patients with STUMP to prevent mortality and long-term sequelae of this disease.
